# Six-Minute Walk Test as a Predictor for Outcome in Children with Dilated Cardiomyopathy and Chronic Stable Heart Failure

**DOI:** 10.1007/s00246-016-1536-y

**Published:** 2016-12-01

**Authors:** Susanna L. den Boer, Daniël H. K. Flipse, Marijke H. van der Meulen, Ad P. C. M. Backx, Gideon J. du Marchie Sarvaas, Arend D. J. Ten Harkel, Gabriëlle G. van Iperen, Lukas A. J. Rammeloo, Ronald B. Tanke, Willem A. Helbing, Tim Takken, Michiel Dalinghaus

**Affiliations:** 1 Division of Pediatric Cardiology, Sophia Children’s Hospital, Erasmus University Medical Center, Dr. Molewaterplein 60, P.O. Box 2060, 3000 CB Rotterdam, The Netherlands; 2 Division of Pediatric Cardiology, Emma Children’s Hospital, Academic Medical Center, Amsterdam, The Netherlands; 3 Division of Pediatric Cardiology, Beatrix Children’s Hospital, University of Groningen, Groningen, The Netherlands; 4 Division of Pediatric Cardiology, Department of Pediatrics, Leiden University Medical Center, Leiden, The Netherlands; 5 Division of Pediatric Cardiology, Wilhelmina Children’s Hospital, University Medical Center Utrecht, Utrecht, The Netherlands; 6 Division of Pediatric Cardiology, Department of Pediatrics, Free University Medical Center, Amsterdam, The Netherlands; 7 Division of Pediatric Cardiology, Department of Pediatrics, Radboud University Medical Center, Nijmegen, The Netherlands; 8 Child Development and Exercise Center, Wilhelmina Children’s Hospital, University Medical Center Utrecht, Utrecht, The Netherlands

**Keywords:** Dilated cardiomyopathy, Pediatrics, Exercise test, 6-minute walk test, Prognosis

## Abstract

Cardiopulmonary exercise testing is an important tool to predict prognosis in children and adults with heart failure. A much less sophisticated exercise test is the 6 min walk test, which has been shown an independent predictor for morbidity and mortality in adults with heart failure. Therefore, we hypothesized that the 6 min walk test could be predictive for outcome in children with dilated cardiomyopathy. We prospectively included 49 children with dilated cardiomyopathy ≥6 years who performed a 6 min walk test. Median age was 11.9 years (interquartile range [IQR] 7.4–15.1), median time after diagnosis was 3.6 years (IQR 0.6–7.4). The 6 min walk distance was transformed to a percentage of predicted, using age- and gender-specific norm values (6MWD%). For all patients, mean 6MWD% was 70 ± 21%. Median follow-up was 33 months (IQR 14–50). Ten patients reached the combined endpoint of death or heart transplantation. Using univariable Cox regression, a higher 6MWD% resulted in a lower risk of death or transplantation (hazard ratio 0.95 per percentage increase, *p* = 0.006). A receiver operating characteristic curve was generated to define the optimal threshold to identify patients at highest risk for an endpoint. Patients with a 6MWD% < 63% had a 2 year transplant-free survival of 73%, in contrast to a transplant-free survival of 92% in patients with a 6MWD% ≥ 63% (*p* = 0.003). In children with dilated cardiomyopathy, the 6 min walk test is a simple and feasible tool to identify children with a higher risk of death or heart transplantation.

## Introduction

Cardiopulmonary exercise testing (CPET) is an important tool which is used to predict prognosis in adults with heart failure [1–3]. Currently, heart transplantation is recommended in patients with a peak oxygen uptake (VO_2_) < 12–14 ml/kg/min, depending on whether patients tolerate beta-blockers or not. In women and in younger patients (<50 years), it has been suggested that those with a peak VO_2_ < 50% of predicted need to be considered for transplantation [4]. Similarly, for children a peak VO_2_ < 50% of predicted for age and sex has been accepted as a Class I indication for heart transplantation (level of evidence C) [5]. More recently, Giardini et al. [6] studied ambulatory children with dilated cardiomyopathy (DCM) and demonstrated that a peak VO_2_ ≤ 62% of predicted was associated with a 10 times higher risk of death and urgent transplantation than a peak VO_2_ > 62%. Although these results are of great importance for heart failure management in children, the use of CPET has some important limitations. It is time-consuming and demanding for patients with severe heart failure. Moreover, it requires sophisticated equipment and specially trained staff. A much less sophisticated exercise test is the 6 min walk test (6MWT). In adults with heart failure, this simple exercise test has been shown an independent predictor for morbidity and mortality [7, 8]. Furthermore, in children with pulmonary hypertension, the 6MWT has been used to indicate disease severity as predictor for death and transplantation and to measure treatment effects [9, 10]. Notably, an advantage of the 6MWT is that it can be performed starting from the age of 6 years.

In the present study, we test the hypothesis that the 6MWT is predictive for the endpoint of death or heart transplantation in children with DCM.

## Methods

This prospective study was approved by the institutional review boards of all seven participating centers. All parents and patients ≥ 12 years gave their written informed consent.

Patients (≥6 years) diagnosed with DCM or followed-up from November 1, 2010, until July 1, 2015, were asked to participate in this study. DCM was defined as the presence of impaired systolic function (fractional shortening [FS] ≤ 25%) and left ventricular (LV) dilation (LV end-diastolic dimension [LVEDD] z-score >+2 for body surface area). DCM could be idiopathic or secondary to other causes. Patients with structural heart defects or neuromuscular disease were excluded.

### Six-Minute Walk Test

At enrollment, all patients performed a 6 min walk test (6MWT) on an 8 m track in a straight corridor. Patients were instructed to walk back and forth on a self-chosen walking speed; running was not allowed. The objective of the test was to walk as far as possible within 6 min. If needed, patients were allowed to slow down the pace or to stop, but were encouraged to resume walking as soon as they were able to. All patients got the same instructions and encouragement at regular intervals, according to the guideline of the American Thoracic Society [11]. During the test, the number of laps was counted. The lap that was partially completed at the end of the test was measured and added to the total distance. The distance walked during 6 min (6MWD) was compared with gender-specific norm values as reported by Geiger et al. [12] using height and age in their regression equations. A percentage of predicted (6MWD%) was calculated by dividing the patients’ 6MWD by the patients’ predicted 6MWD, and multiplying this by 100%. Transcutaneous oxygen saturation and heart rate were recorded before and immediately after the test. Using a maximum heart rate of 200 beats per minute (bpm) for all ages, [13] the heart rate immediately after the test was transformed to a percentage of the maximum.

At the same visit, patients’ demographics were recorded. Height and weight were measured. N-terminal pro-B-type natriuretic peptide (NT-proBNP) was determined. A complete and standardized echocardiogram was performed and analyzed off-line by one investigator (SdB). M-mode of the parasternal long-axis was used to measure LVEDD and LV end-systolic dimension and subsequently, FS was calculated. LV ejection fraction was calculated using Simpson’s biplane method. The 6MWT results were not relayed to the treating physicians and were only stored in the research database. Results of the 6MWT were not used in clinical decisions. The study endpoint was death or heart transplantation. Listing strategies followed the American Heart Association guidelines [5]. Follow-up continued until September 15, 2015.

### Statistical Analysis

Continuous variables are displayed as mean ± standard deviation (SD) if normally distributed, and as median with interquartile range (IQR) if non-normally distributed. NT-proBNP results were log-transformed because of the non-normal distribution. Means across groups were compared using independent sample *t* test. Transplant-free survival was estimated with the Kaplan–Meier method, and 95% confidence intervals (CI) were calculated using Greenwood’s formula. The log-rank test was used to determine statistical significance between two transplant-free survival curves. A receiver operating characteristic curve was generated using 6MWD% to define the optimal threshold to identify patients at highest risk for an endpoint. Univariable Cox regression analysis was used to test the predictive value of 6MWD% as a continuous and as a binominal variable based on the threshold. Other potential risk factors were also tested with univariable Cox regression. The number of variables allowed for multivariable analysis was set at the number of events divided by ten. All analyses were performed using IBM SPSS Statistics for Windows, version 21.0 (IMB Corp, Armonk, NY). Testing was performed two-sided, and statistical significance was defined as *p* < 0.05.

## Results

During the 4.5 years of the study, 64 patients were eligible, 56 (88%) gave written informed consent. Seven patients were too ill and reached the endpoint before they performed a 6MWT. In total, 49 patients performed a 6MWT, at a median age of 11.9 years. The median time since diagnosis was 3.6 years; 9 (18%) were included within 3 months of diagnosis, 35 (71%) more than 1 year after diagnosis. Patients were on optimal pharmacological therapy, 94% used angiotensin converting enzyme-inhibitors and 78% used beta-blockers (Table 1). In patients included within 1 year of diagnosis, the use of beta-blockers was somewhat lower and NTproBNP was higher, but overall the profile of those included within and after 1 year of presentation was comparable (Table 1.)

**Table 1:** Characteristics of children with dilated cardiomyopathy and 6 min walk test result

	*n* = 49	Time since diagnosis <1 year *n* = 14	Time since diagnosis >1 year *n* = 35
Gender, male, *n* (%)	26 (53)	5 (36)	21 (60)
Age, years	11.9 (7.4–15.1)	12.1 (8.6–16.0)	11.8 (8.3–14.1)
Time since DCM diagnosis, years	3.6 (0.6–7.4)	0.1 (0.1–0.5)	6.2 (3.1–9.2)
Cause of DCM, *n* (%)			
Idiopathic	24 (49)	6 (43)	18 (51)
Myocarditis	7 (14)	0 (0)	7 (20)
Other	18 (37)	8 (57)	10 (29)
Medication used, *n* (%)			
Beta-blocker	38 (78)	9 (64)	29 (91)
ACE-inhibitor	46 (94)	14 (100)	32 (91)
Spironolactone	27 (55)	8 (57)	19 (54)
Loop diuretics	25 (51)	8 (57)	17 (49)
Digoxin	8 (16)	1 (7)	7 (20)
NT-proBNP (pmol/L)	221 (51–555)	451 (248–735)	102 (32–472)
LVEDD z-score	5.0 ± 3.2	4.7 ± 2.1	5.2 ± 3.5
Fractional shortening, %	18 ± 6	15 ± 7	19 ± 6
LV ejection fraction, %	33 ± 12	25 ± 11	36 ± 12
Endpoint, *n* (%)	10 (20)		
Death	0		
Heart transplantation	10 (100)	3	7
Follow-up since 6MWT, months	33 (14–50)	24 (12–31)	45 (14–53)
6MWD, m	448 ± 144	401 ± 88	466 ± 88
6MWD%, %	70 ± 21	62 ± 15	73 ± 22

Continuous variables are represented as mean ± SD if normally distributed and as median (IQR) if non-normally distributed

Cause of DCM—“Other” includes Anthracycline cardiomyopathy (5), familial (5); non-compaction cardiomyopathy (3), ischemia (2), arrhythmia (2)

*6MWD* indicates 6 min walk distance, *6MWD%* 6 min walk distance as % of predicted, *ACE* angiotensin converting enzyme, *DCM* dilated cardiomyopathy, *LVEDD* left ventricular end-diastolic dimension

### Six-Minute Walk Test Results

Mean 6 min walk distance as percentage of predicted (6MWD%) was 70 ± 21%. There was no difference in 6MWD% in patients included within or more than 1 year after presentation reaching an endpoint (53 vs. 52%), or not reaching an endpoint (64 vs. 75%). Patients included >1 yr after presentation reaching an endpoint scored lower (52 vs. 75% *P* < 0.05); the group <1 year after presentation was too small to test statistically. Two patients (4%) stopped walking during the test. The distance that they covered was accepted as final result of the 6MWT. One patient, 6.8 years old, complained about chest pain and stopped walking at 3 min. The second patient, 6.0 years old, stopped several times, because she felt too tired. She had severe heart failure and underwent heart transplantation 4 months after the test. No other complications were registered during the tests. The heart rate before and immediately after the test is displayed in Table 2. Pre-6MWT, the mean heart rate in patients not taking beta-blockers was significantly higher (98 ± 12 bpm) than in patients with beta-blocker therapy (85 ± 15 bpm, *p* = 0.02). No differences in age were found between patients with and without beta-blocker therapy. Post-6MWT, the mean HR was 124 ± 18 bpm, which was 62% of the maximum. Patients not taking beta-blockers had a mean heart rate of 66 ± 8% of the maximum, while this was 61 ± 9% in those using beta-blockers (*p* = 0.08).

**Table 2:** Heart rate and oxygen saturation pre- and post-6-min walk test in 49 children with dilated cardiomyopathy

Age range (years)	n*	SaO_2_		n*	Heart rate (bpm)		% of max
		Pre-6MWT	Post-6MWT		Pre-6MWT	Post-6MWT	Post-6MWT
6–8	9	99 (98, 100)	98 (95, 98)	11	94 ± 13	132 ± 16	66 ± 8
8–12	10	99 (98, 100)	98 (97, 98)	11	88 ± 13	125 ± 20	63 ± 10
12–15	12	98 (98, 99)	97 (97, 98)	12	85 ± 15	121 ± 16	61 ± 8
15–18	10	98 (97, 99)	97 (96, 98)	10	85 ± 19	117 ± 18	58 ± 9

* Eight cases were missing for SaO_2_, five for heart rate

*6MWT* indicates 6 min walk test, *bpm* beats per minute, *SaO*_*2*_ percutaneous oxygen saturation

### Outcome

During the study, ten patients reached the endpoint of heart transplantation, none of the patients died. In these patients, NTproBNP and LVEDD z-score was higher and FS and EF were lower (Table 3) underscoring the severity of their heart disease at the time of the 6MWT. At the time of listing, 80% had a Class I indication for heart transplantation, three were admitted on inotropes, one intermittently on inotropes, and the others had severe limitations in daily life, in many more than 50% absence from school. At the time of transplantation, the three patients on inotropes all had progressed to require MCS, all others were at home. The time on the waiting list varied from 1 day to more than 3 years. Of all study patients, the median follow-up time was 33 months. This resulted in a 1 year transplant-free survival of 89% (95% CI 80–98) and 2 year transplant-free survival of 84% (95% CI 74–95). The median time from diagnosis to transplantation was 3.9 years (IQR 2.4–8.2); the three patients that were tested <1 year of presentation underwent transplantation after 1.7–2.4 years. The seven patients who were too ill to perform a 6 MWT before they reached an endpoint had a median time from DCM diagnosis to the endpoint of 31 days (IQR 16–75). Of them, three died and four were transplanted (two of whom were on mechanical circulatory support).

**Table 3:** Univariable analysis of potential risk factors for death or heart transplantation in 49 children with dilated cardiomyopathy

Potential risk factors for outcome	Primary endpoint (*n* = 10)	No primary endpoint (*n* = 39)	Hazard ratio (95% CI)	*p* value
6MWD%, %	55 ± 23	74 ± 19	0.95 (0.91–0.99)	0.006
6MWD% < 63%, *n* (%)	8 (80)	12 (31)	7.51 (1.59–35.5)	0.011
Age at DCM diagnosis ≥ 6 years, *n* (%)	6 (60)	18 (46)	2.02 (0.57–7.24)	0.27
NT-proBNP (pmol/L)	569 (364–742)	99 (32–420)	30.1 (1.9–479)^a^	0.016
LVEDD z-score	7.0 ± 4.2	4.5 ± 2.7	1.25 (1.02–1.53)	0.032
Fractional shortening, %	14 ± 5	19 ± 6	0.86 (0.75–0.97)	0.016
LV ejection fraction, %	21 ± 8	36 ± 11	0.88 (0.81–0.96)	0.003

^a^ Hazard ratio for log10(NT-proBNP) because data is skewed. Meaning, 10-fold increase in NT-proBNP corresponds with a 30 times higher risk of death or heart transplantation

*6MWD%* indicates 6 min walk distance as % of predicted, *DCM* dilated cardiomyopathy, *LVEDD* left ventricular end-diastolic dimension

### Risk Factors for Outcome

**Fig. 1:**
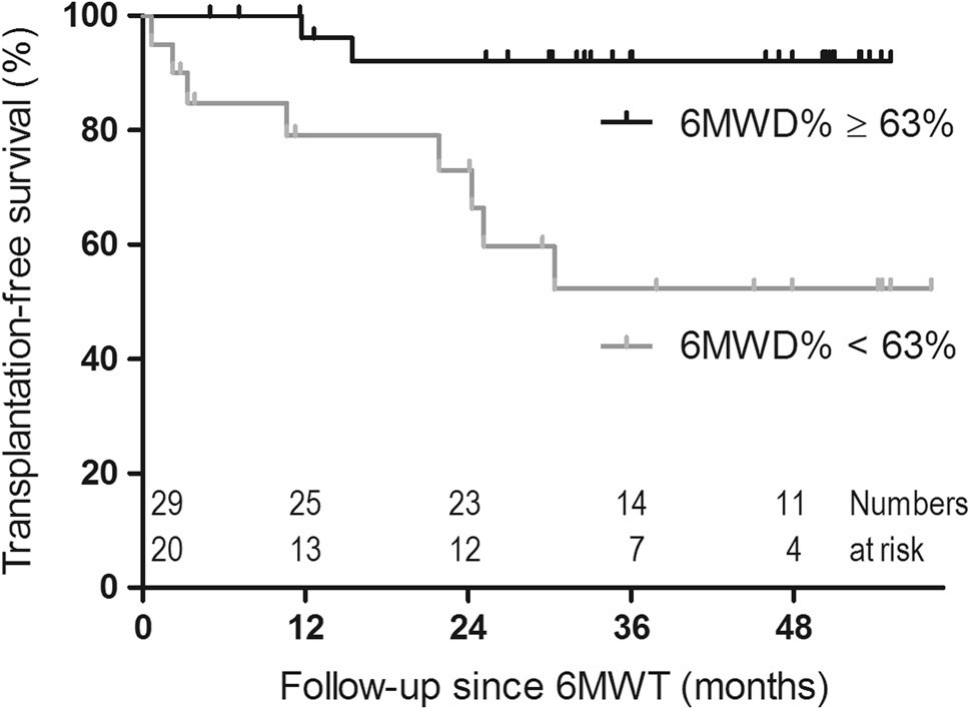
Transplant-free survival curves of DCM patients with a 6 min walking distance ≥63% of predicted and of those <63% of predicted. 6MWD% indicates 6 min walk distance as percentage of predicted; 6MWT, 6 min walk test

The 6MWD% was a significant predictor for the risk of death or transplantation (hazard ratio 0.95 per % of predicted, 95% CI 0.91–0.99, *p* = 0.006). Thus, every % decrease in the 6MWD%, gave a 5% increase in the risk of death or transplantation. A 6MWD% < 63% identified patients at highest risk for an endpoint (sensitivity 80% and specificity 69%, area under the curve 0.76, 95% CI 0.57–0.95, *p* = 0.01). One-year transplant-free survival was 79% (95% CI 61–98), and 2 year transplant-free survival was 73% (95% CI 53–93), in contrast to patients with a 6MWD% ≥ 63% in whom 1 year transplant-free survival was 96% (95% CI 89–100), and 2 year transplant-free survival was 92% (95% CI 82–100, *p* = 0.003, Fig. 1). Univariate analysis of other potential risk factors showed that a higher NT-proBNP and LVEDD z-score and a lower LV SF and EF were significantly predictive for the risk of death or transplantation (Table 3). The number of events in this study did not allow multivariable analysis.

## Discussion

In this prospective study, we demonstrated that the 6 min walking distance expressed as a percentage of predicted was associated with prognosis in children with DCM. A higher total distance walked in 6 min resulted in a lower risk of death or heart transplantation. This is important because markers that identify children with a good or bad prognosis during follow-up are essential to guide clinical management in this patient group.

The study group had a mean age of 11.9 years and a median time since DCM diagnosis of 3.6 years, representing children with chronic heart failure. Although DCM has high incidence rates at young age, [14] the age-restriction for the test resulted in a relatively older group. An earlier study investigating the predictive value of CPET included patients with a mean age of 13.5 years, reflecting the somewhat older age at which CPET can be performed [6].

All study patients who reached an endpoint, underwent transplantation. Earlier outcome results of children with DCM have shown that after the first year of diagnosis, the large majority of children reaching an endpoint undergo transplantation [15, 16]. The median time after diagnosis in our study group was 3.6 years, which may explain the relatively high number of transplantations compared to deaths. Furthermore, our results indicate that children that were included early after presentation and who were able to perform a 6MWT represent a less sicker part of the population. The children that were unable to perform a 6MWT all reached an endpoint early after presentation, while those who could perform a 6MWT and reached an endpoint, underwent transplantation 2 years after presentation. The 6MWT has been done for research purposes only, and the results were not relayed to the treating physicians. Consequently, the 6MWT results have not influenced transplantation decisions. Therefore, the 6MWT can be used as predictor for deterioration of the disease during the follow-up of DCM.

After 4.5 years of patient inclusion and almost 3 years of follow-up, the number of endpoints was limited and multivariable analysis was not allowed. The predictive value of other variables on outcome was assessed to characterize our patient population and we showed that besides the 6MWD%, NT-proBNP, LVEDD z-score, LVEF and LVFS were predictive. Considering the univariable results after correcting for multiple testing, a *p* value of <0.0071 would be significant. At this level, the 6MWD% and LVEF would remain significant. LVEF has been shown to be predictive in earlier pediatric reports and has also been valuable in predicting prognosis in adults [17, 18]. The 6MWD% is a new finding in children and is likely an easily accessible and valuable tool in predicting outcome in this patient group. We suspect that serial testing may enhance the predictive value of the 6MWT in this chronic heart failure population, but more data and more endpoints need to be accumulated to test that assumption. Further investigation in a larger population needs to establish whether the 6MWT holds as an independent marker in multivariable analysis.

Exercise testing is one of the cornerstones in predicting prognosis in ambulatory heart failure patients [1–4]. Specifically, peak VO_2_ measured with CPET has a prominent role in heart transplantation guidelines [4, 5]. A peak VO_2_ < 12–14 ml/kg/min in adults and <50% of predicted in children has been accepted as a Class I indication for heart transplantation. In addition, the 6MWT has been shown to be a predictor for mortality and hospitalization for adults with chronic heart failure [7, 8, 19, 20]. Although it seems that CPET results are superior to 6MWT results in order to predict prognosis, [19, 21] the 6MWT has major advantages, such as its simplicity and its inexpensiveness. Therefore, it is particularly relevant as follow-up and screening tool.

As CPET measures maximal exercise capacity, the 6MWT measures submaximal exercise capacity. This is also reflected by the maximal heart rates reached with the 6MWT. Post-6MWT, we found a mean heart rate of 62% of the maximum, while this was around 70% in the norm population [12]. In patients with heart failure chronotropic incompetence has been described as a result of beta-receptor down-regulation and desensitization of the beta-receptors [22]. Moreover, beta-blocker therapy may affect the ability to reach the maximal heart rate as predicted for age. A prevalence of 37% of chronotropic incompetence has been described in a cohort of children with chronic DCM [6]. Thus, maximal heart rates may well have been lower in patients in our study. By assuming a maximal heart rate of 200 bpm, the submaximal heart rate as percentage of the maximum may have been underestimated.

### Study Limitations

We studied almost all eligible children (88%) in the Netherlands during a 4 year period, but the sample size was small. Furthermore, we did not measure maximal exercise capacity (CPET) in our cohort. Nevertheless, we were able to show that the 6MWT is feasible and easy to perform in children with DCM and is a valuable predictor for outcome. Therefore, 6 min walk testing should be implemented in this patient group to estimate the patients’ risk and to analyze these results in future studies using multivariate analysis. Furthermore, we used an 8 m track to obtain the results, rather than a 30 m track as is recommended in the American Thoracic Society guidelines [11]. Because 6MWTs were performed at seven different centers and not all had a quiet 30 m corridor available, we decided to use an 8 m track in all centers to minimize test variation. We recognize that the use of a shorter track has led to more turning points and may have led to a small reduction in the total distance walked. However, because patients walked slower than healthy children, this would have had less impact. To interpret our recommended cutoff to a population walked on a 30 m track, the cutoff may become slightly higher. Finally, the cutoff is based on our dataset and may differ in another population. Therefore, it needs to be validated externally.

## Conclusions

In the present study, we demonstrate that the 6 min walk test is feasible and has predictive value in children with DCM ≥ 6 years. A lower distance walked, expressed as percentage of predicted, was associated with a higher risk of death or heart transplantation. A cutoff of 6MWD% < 63% identified patients at highest risk of an endpoint.
